# Laryngectomy in Elderly Patients: A Case Series and Review of the Literature

**DOI:** 10.7759/cureus.49294

**Published:** 2023-11-23

**Authors:** Leyla Ozbek, Peter Lion, Ankit Patel, Jonathan Hughes, Kuntal Shah, Paul Stimpson

**Affiliations:** 1 Otolaryngology, University College Hospital, London, GBR; 2 Head and Neck Surgery, University College Hospital, London, GBR

**Keywords:** salvage total laryngectomy, laryngeal pathology, head and neck neoplasms, squamous cell carcinoma (scc), total laryngectomy

## Abstract

Background and objective

Patients over the age of 75 years make up 20% of the head and neck cancer population, which is a relatively under-represented patient cohort in clinical literature. To our knowledge, there are no studies evaluating the outcomes of laryngectomy in patients aged over 75 years, which prompted us to present this unique series.

Methods

We reviewed departmental records at the University College Hospital, London over a 10-year period, and identified a total of 18 patients over the age of 75 years who underwent total laryngectomy for squamous cell carcinoma. We evaluated the demographic, clinical, and histopathologic features and outcomes for each patient.

Results

The age of the cohort ranged from 75 to 90 years, with a mean age of 79.8 years. All patients had a Charlson Comorbidity Index (CCI) score of 3 or more (due to age), with a mean of 4.7, and a maximum score of 8 for two patients. Length of inpatient stay varied significantly, ranging from 20 to 149 days, with a mean of 46 days. We identified 14 patients who underwent laryngectomy prior to September 2017, in whom the five-year survival was 21.4%. The three-year survival rate for all patients was 22.2%. In bivariate analysis, advanced age at surgery positively correlated with increased length of hospital admission and increased incidence of complications, although these results were not statistically significant (*p*<0.05).

Conclusions

Our study highlights the significance of the impact of age and comorbidities on postoperative outcomes and sheds light on the unique challenges faced by an ageing population. Careful consideration must be made in terms of appropriate patient selection, and clinicians must offer a robust and tailored approach to elderly care.

## Introduction

Around 12,400 new head and neck cancer cases are reported in the United Kingdom (UK) annually, and the incidence has increased by 34% since the early 1990s [1]. Of note, 20% of head and neck cancers are diagnosed in those aged over 75 years, with 10% being in those over 80 years [2]. It is estimated that roughly 20% of the UK population will be over 75 years of age by 2030 [3], making this an ever-growing patient cohort. The World Health Organisation (WHO) defines an elderly person as someone aged 65 years or over; however, the National Institute on Ageing offers a more robust definition, classifying patients as “young old” (65-74 years), “older old” (75-85 years) and “oldest old” (>85 years) [4].

The rapid change in the demographics of the developed world [5] has led to an increased burden on health services, rendering the appropriate care of elderly patients an increasingly pertinent issue. Elderly patients may not be offered treatments as aggressively as their younger counterparts, leading to undertreatment and poorer outcomes. There is often a lack of good quality evidence in the form of clinical trials to help guide clinicians in the management of this patient demographic, as many elderly patients are excluded from research due to age alone [6].

Although papers outlining laryngectomy and other surgical interventions for laryngeal cancer in the over-65-year age group do exist in the literature, to our knowledge, there is a scarcity of such data regarding patients in the “older old” category of over 75 years [4]. In light of this, we present this unique series that aims to shed light on the background and outcomes of such patients, a cohort that clinicians will increasingly be required to manage in the coming years.

This article was previously presented as a poster at the British Academic Conference of Otorhinolaryngology, Birmingham, UK, on February 15, 2023.

## Materials and methods

Study setting and inclusion criteria

We reviewed departmental records at the University College Hospital in London over a 10-year period, from 2009 to 2019, to identify patients aged 75 years or more who underwent a total laryngectomy. The inclusion criteria were any patient over the age of 75 years undergoing total laryngectomy; these patients were identified from a database maintained by one of the authors. Patients undergoing simultaneous additional procedures, such as neck dissection, were also included. Patients with histology other than squamous cell carcinoma were excluded.

Data collection

We retrospectively collated data on comorbidities, TNM staging, and surgery performed, by reviewing the hospital's electronic records. The Charlson Comorbidity Index (CCI) score, which predicts 10-year survival in patients with multiple comorbidities, was calculated for each patient; it includes the patient’s age and the presence of a number of key illnesses. The Clavien-Dindo classification of complications for each patient was also included, which is a widely used scoring system for classifying adverse events following surgery. Grade I pertains to any deviation from the normal surgical course, which includes the administration of medications such as analgesics. Grade II relates to complex drug treatments, such as blood transfusions or intravenous antibiotics, and grade III involves any surgical or radiological intervention. Grade IV describes single or multiorgan failure, and grade V classifies the death of the patient. We also reviewed the postoperative outcomes including length of inpatient stay, voice rehabilitation, nutrition, length of follow-up, recurrence rate, and survival.

## Results

We identified a total of 18 patients, of which 16 (88.9%) were male and two (11.1%) were female. Their ages ranged from 75 to 90 years, with a mean age of 79.8 years. The patients suffered from a range of comorbidities, which were scored using the CCI score - all patients had a score of 3 or more (due to age), with a mean of 4.7, and a maximum score of 8 for two patients. One patient had T1 disease, and two patients had T2 disease, and all three of them were salvage laryngectomy cases who had undergone previous radiotherapy. Three patients had T3 disease, and the remaining 13 (72.2%) patients had T4 disease. In total, seven (38.8%) patients were salvage laryngectomy cases. All patients underwent total laryngectomy, with nine patients undergoing neck dissection (unilateral or bilateral). Among the patients undergoing salvage surgery, one patient underwent reconstruction via gastric pull-up while three patients had augmentation of their reconstruction with a pedicled flap. The data on patient demographics, disease characteristics, and treatment are presented in Table 1.

**Table 1:** Patient demographics, disease characteristics, and treatment

Characteristics	Number of patients (%)
Sex	
Male	16 (88.8)
Female	2 (11.2)
Charlson Comorbidity Index score	
3	3 (16.7)
4	8 (44.4)
5	2 (11.2)
6	1 (5.5)
7	2 (11.2)
8	2 (11.2)
Primary tumour site	
Hypopharynx	2 (11.2)
Larynx	16 (88.8)
T classification	
T1	1 (5.5)
T2	2 (11.2)
T3	2 (11.2)
T4	13 (72.10
N classification	
N0	15 83.3
N2c	2 (11.2)
N3	1 (5.5)
Type of surgery	
Primary	11 (61.1)
Salvage	7 (38.9)
Postoperative Clavien-Dindo classification	
2	15 (83.5)
3	1 (5.5)
3b	1 (5.5)
5	1 (5.5)
Voice rehabilitation	
Tracheo-oesophageal puncture	14 (77.8)
Nil	4 (22.2)
Enteral feeding	
Yes	2 (11.2)
No	16 (88.8)
Postoperative recurrence	
Yes	4 (22.2)
No	14 (77.8)

The length of inpatient stay varied greatly, ranging from 20 to 149 days, with a median of 38 days. Complications were graded according to the Clavien-Dindo classification, with all patients scoring a minimum of 2, and six (33%) patients suffering from notable complications, including one patient who had a cardiac arrest in the postoperative period and later died while still an inpatient. Of note, 14 of the patients successfully regained their voice using a voice prosthesis via tracheo-oesophageal puncture. The remaining four patients were unable to undergo surgical voice rehabilitation. All but two patients resumed normal oral intake, with the remaining patients requiring gastrostomy for nutrition. 

As the length of follow-up varied among patients, we calculated the five-year survival for patients undergoing laryngectomy in 2017 or prior, and the three-year survival for all patients; 14 patients underwent laryngectomy prior to September 2017, and their five-year survival rate was 21.4%; the three-year survival for all 18 patients was 22.2%. The salvage group had a three-year survival rate of 14% while it was 27% in the primary group. Interestingly, the five-year survival rate of salvage laryngectomies was found to be zero. 

The mean CCI score for the four patients still alive at three years was 3.75, while it was 5.1 for those who died. Bivariate analysis was performed using the Pearson correlation coefficient, which revealed that advanced age at surgery positively correlated with increased length of hospital admission and increased incidence of complications (Table 2), although these results were not statistically significant. Interestingly, as comorbidities increased, the length of stay decreased, although this data was skewed by one outlying variable; once this outlier was removed, there was a positive correlation between higher CCI score and increased length of stay, although these results were not statistically significant either. As comorbidities increased, the complication rate was found to decrease as well (*p*=0.67). Pearson chi-square testing did find a statistically significant association between male gender and higher CCI score (*p*=0.003), as well as increased Clavien-Dindo score for postoperative complications (*p*<0.001) (SPSS Statistics for Mackintosh, Version 29.0., IBM Corp., Armonk, NY).

**Table 2:** Correlation coefficients between patient variables CCI: Charlson Comorbidity Index

	Age at surgery	CCI score	Length of inpatient stay	Clavien-Dindo
Age at surgery	-	0.464	0.219	0.778
CCI score	0.464	-	-0.402	-0.686
Length of inpatient stay	0.219	-0.402	-	0.683

## Discussion

Age has been shown to have a statistically significant impact on local postoperative complications for patients undergoing total laryngectomy. Indeed, when looking at all general postoperative complications, age again had a statistically significant impact on the incidence of such issues, with the risk of complications two-fold greater in the age group of >65 years [7]. However, it is important to bear in mind that other factors also have a significant impact on postoperative outcomes, with the same study showing that type of surgery, cardiac history, and American Society of Anaesthesiologists (ASA) grade are also pertinent variables and that these concerns are not unique to total laryngectomy; the risk of complications for elderly patients has been shown to be higher in other interventions for laryngeal cancer as well, such as laser cordectomy and radiotherapy [8]. This is certainly a pertinent issue, as complications also increase the length of inpatient stay, and the cost of hospitalisation, apart from delaying the commencement of adjuvant therapies.

However, some studies have shown that age is not associated with the risk of complications or postoperative survival in patients. Clayman et al.'s study showed no increase in complications for patients in the over-80 cohort undergoing ear, nose and throat operations [9], while Teymoortash et al. concluded that disease-free and overall survival is not reduced in patients aged over 65 years undergoing laryngectomy with neck dissection [10]. Indeed, it has been shown that survival outcomes are not different in elderly patients with laryngeal cancer when stratified for disease stage [11]; however, this study encompassed all treatment modalities. Linking this back to our findings, we were unable to uncover a statistically significant link between increasing age and increasing risk of postoperative complications among patients. This highlights that age should not be the sole deciding factor in the suitability of patients for major surgery.

Despite the undeniable impact of age on postoperative outcomes, comorbidities have been shown to be the main risk factor for mortality and morbidity in head and neck operations [12]. While there is certainly a correlation between age and postoperative complications, as discussed previously, this link remains more tenuous in comparison to the correlation between comorbidities and complications [10]. In our study, however, we were unable to find a statistically significant link between the increasing presence of comorbidities and worsening postoperative outcomes; this is replicated in another study involving older patients undergoing total laryngectomy [10] but remains generally at odds with what is found in the literature. 

The ageing population faces a unique set of challenges, which must be acknowledged by clinicians in order to provide well-rounded care for these patients. Symptoms of cancer can be less evident in older patients [13], although this may not be the case in those presenting with laryngeal malignancy [10]. Elderly patients frequently receive sub-optimal treatment, with full surgical intervention being less likely to be offered. Findings from one centre revealed that treatment delivered to elderly patients presenting with a head and neck tumour complied with local guidelines in less than 50% of cases [2]. This highlights the need for a specialised treatment plan for elderly patients, particularly as the number of older patients being managed within our healthcare systems is rising. However, achieving this is made difficult by the lack of clinical research specifically assessing patients in their later years - frail patients are less likely to be included in clinical trials, and hence information remains relatively scarce [8]. Trials often tend to focus on survival whereas there is an even greater need for focusing on the quality of life alongside survival in our elderly patients [14].

Expected survival duration following operative interventions is an important factor to consider when undertaking a treatment as invasive as total laryngectomy. Several factors may likely affect postoperative survival in patients, including tumour staging and ongoing carcinogen exposure [15]. In patients with hypopharyngeal cancer, a higher disease stage or grade results in a lower five-year survival [16]. Age has been shown to have a more moderate effect on postoperative outcomes - a study of 133 patients with laryngeal carcinoma showed no significant difference in five-year survival following laryngectomy between those aged over 60 years (39% survival) and younger patients (50% survival) [15]; however, these results were found to be more favourable compared to our five-year survival rate of 21%. Although further research is needed regarding laryngectomy, elderly patients undergoing surgical treatment for hepatocellular carcinoma had fewer ‘years of life’ lost compared to their younger counterparts, meaning that despite shorter postoperative survival, they had more relative years of life gained [17].

There are a number of learning points to be gathered regarding the decision to proceed with laryngectomy in elderly patients; Shi et al. [13] have suggested that as long as patients are adequately prepared, surgical intervention still remains the most viable treatment option for most elderly patients, and some authors suggest using a Comprehensive Geriatric Assessment score to assist in their decision making, rather than ASA grade as this has not shown to accurately predict postop morbidity [2,18]. The best outcome is likely to be achieved by taking into account numerous variables, including the incidence of comorbidities, the functional status, the characteristics of the disease, and the patient’s expectations regarding the outcome, as opposed to relying on age alone [6].

This study has a few limitations, primarily our small cohort size, which could be attributed to the rarity of laryngectomy in this age group. Our findings may also have been influenced by retrospective selection bias, as it is possible that patients were selected for laryngectomy because they were thought to be suitable candidates; hence, these findings may not be representative of the elderly population as a whole.

## Conclusions

Although there are a number of studies evaluating the outcomes of total laryngectomy in patients over the age of 65 years, there is a scarcity of such data related to the age group of >75 years. Our study contributes valuable data to this under-researched field, thereby helping to improve our understanding of surgical outcomes in this patient cohort. This is particularly important given the rapid expansion of this age group. We aimed to bring to light the unique challenges faced by an ageing population and show that careful consideration must be made towards appropriate patient selection, with clinicians offering a robust and tailored approach to elderly care. Despite the undeniable impact of age on postoperative outcomes, comorbidities have been shown to be the main risk factor for mortality and morbidity in head and neck operations; this highlights the learning point that age should not be the sole deciding factor in the suitability of patients for major surgery, and all patient-related variables should be considered in their entirety.
